# Unlikely Remedy: Fungicide Clears Infection from Pathogenic Fungus in Larval Southern Leopard Frogs (*Lithobates sphenocephalus*)

**DOI:** 10.1371/journal.pone.0043573

**Published:** 2012-08-17

**Authors:** Shane M. Hanlon, Jacob L. Kerby, Matthew J. Parris

**Affiliations:** 1 Department of Biological Sciences, University of Memphis, Memphis, Tennessee, United States of America; 2 Department of Biology, University of South Dakota, Vermillion, South Dakota, United States of America; Imperial College Faculty of Medicine, United Kingdom

## Abstract

Amphibians are often exposed to a wide variety of perturbations. Two of these, pesticides and pathogens, are linked to declines in both amphibian health and population viability. Many studies have examined the separate effects of such perturbations; however, few have examined the effects of simultaneous exposure of both to amphibians. In this study, we exposed larval southern leopard frog tadpoles (*Lithobates sphenocephalus*) to the chytrid fungus *Batrachochytrium dendrobatidis* and the fungicide thiophanate-methyl (TM) at 0.6 mg/L under laboratory conditions. The experiment was continued until all larvae completed metamorphosis or died. Overall, TM facilitated increases in tadpole mass and length. Additionally, individuals exposed to both TM and *Bd* were heavier and larger, compared to all other treatments. TM also cleared *Bd* in infected larvae. We conclude that TM affects larval anurans to facilitate growth and development while clearing *Bd* infection. Our findings highlight the need for more research into multiple perturbations, specifically pesticides and disease, to further promote amphibian heath.

## Introduction

Anthropogenic perturbations, such as pesticides, often act as stressors for non-target organisms. Pesticides are common contaminants that enter aquatic systems through runoff, overspray, or pesticide drift [1], [2]. Thus, ecologists are charged with examining the manner in which contaminants affect non-target aquatic organisms such as amphibians.

Under realistic conditions, agrochemicals such as pesticides are applied multiple times throughout a growing season and non-target organisms commonly experience reoccurring exposure [3], [4]. Depending upon the half-life of the specific chemical, the reapplication of pesticides may not allow for its natural breakdown into less harmful products before another exposure occurs. When pesticide dosages are lethal, the number of exposures is inconsequential. However, at sublethal levels, repeatedly exposed individuals may not have an opportunity to recover from an initial dose. Accordingly, ecologists have examined the differences between “pulse” (a single, initial dose) and “press” (multiple exposures over time) treatments for many years [5]–[7]. Many pulse experiments have illustrated the ability of an individual, community, or both to rebound from a single exposure [4], [8]. One might conclude that using press experiments in which individuals are subjected to the reoccurring pressure of pesticide exposure to be the test that most closely reflects patterns of exposure experienced by most affected organisms [9].

One pesticide that is applied worldwide is the fungicide thiophanate-methyl (TM; http://water.usgs.gov/nawqa/pnsp/usage/maps/show_map.php?year=02&map=m5019). TM is a broad-spectrum fungicide that targets mycorrhizal fungi. TM has been marketed as the replacement to benomyl [10]; the most widely used fungicide in the United States until its discontinuation in 2001 [10].

TM is used heavily in the Mississippi River Basin. Bishop et al. [11] assessed fungicide usage in Tennessee and northern Mississippi and concluded that TM was used widely throughout both states. It is reasonable to assume that TM is entering aquatic environments as has been found for other pesticides. However, no research has examined the effects of TM on anurans. This may be due in part to the relatively short half-life of TM [12] and potential breakdown into inert products. However, a byproduct of TM breakdown, carbendazim, has been linked to adverse effects on amphibian larval growth, development, and survival [13]. Even so, with repeat pesticide exposure, runoff, and spraydrift [9], [14], aquatic organisms with prolonged larval periods are likely exposed to TM multiple times through ontogeny, thus decreasing the probability of breakdown into inert components. Multiple studies have examined the effects of an LD_50_ (the lethal dosage required to kill half a population) injected dose of TM [15]–[17]. However, this application method does not serve as a valid proxy for environmental exposure. Accordingly, tests where TM is applied to an organism’s habitat (LC_50_), as opposed to directly into the organism itself, provide a more relevant indication of adverse effects. While natural water sample analysis has not been conducted to determine environmental concentrations of TM, pilot studies reveal that possible LC_50_ values range from 7.5 to 10 mg/L (Hanlon, unpublished data).

Along with the aforementioned chemical factors, organisms are also exposed to additional pressures, such as pathogens. One pathogen that is causing rapid declines in amphibian populations is the emerging infectious disease chytridiomycosis, caused by the pathogenic fungus *Batrachochytrium dendrobatidis* (*Bd*). *Bd* infects keratinized tissues, such as anuran larval mouthparts, reducing their foraging capabilities [18]–[20]. While *Bd* does not generally cause mortality in larvae (as it does in adults), the fungus often impairs growth and developmental rates [21]–[24]. Venesky et al. [20] found that *Bd* altered larval mouthparts, resulting in *Hyla chrysoscelis* (Cope’s treefrog) larvae foraging less efficiently than uninfected individuals. Additionally, Hanlon et al. (unpublished data) found that infected *Hyla versicolor* (gray treefrog) larvae spent significantly more time foraging than uninfected individuals. However, the authors observed no corresponding increases in growth and development with increased foraging. Together, these results indicate that while infected larvae may spend more time foraging than uninfected individuals, they are unable to fully compensate for the deficits in efficiency.

Although much is known about the independent effects of pesticides and *Bd* on amphibians, a limited number of studies have examined the possible interactive effects of these two perturbations. Currently, two research approaches are being developed to test such interactions: 1) testing the effects of contamination on disease independent of hosts, and 2) testing the effects of contamination on disease in amphibian hosts. Studies that examine possible interactions of contaminants and pathogens outside of a host mimic situations prior to or following host infection. *Bd* can persist within the environment (independent of an amphibian host) for up to seven weeks [25], allowing for the possibility of an interaction outside of hosts. Additionally, Hanlon and Parris [26] showed that the pesticides carbaryl, glyphosate, and TM killed *Bd* in culture independent of potential hosts. On the other hand, research such as our current study that examine the interactive effects of *Bd* and a contaminant upon a host mimic a post-infection scenario. Such situations indicate the possibility of interactive effects between *Bd* and pesticides, causing reduced foraging efficiency and likely life history consequences.

While studies have found negative interactive effects of pesticides and pathogens within hosts [27], [28], studies testing for interactions between *Bd* and anti-fungal agents have yielded significantly different results. Many anti-fungal treatments kill *Bd* in culture [29]–[31]. Also, anti-fungals kill *Bd* in hosts [29], [31], [32]. However, the broader impacts of such treatments on non-target organisms are largely unknown and thus prevent addition of such chemicals to natural habitats. While the addition of these chemicals to natural environments is not possible, fungicidal pesticides are applied in great quantities across the United States. Accordingly, we examined the interactive effects of the fungicide TM and *Bd* on larval anurans under laboratory conditions. We predicted that *Bd* exposure would facilitate reductions in growth and TM alone at sublethal levels would have no effect on growth. Also, because TM has been shown to kill *Bd* in culture independent of hosts, we predicted that TM would clear *Bd* infection in individuals exposed to both *Bd* and TM.

## Methods

### Animal Collection and Husbandry

*L. sphenocephalus* eggs were collected from ponds within Shelby Farms Park in Shelby County, TN (35° 9′ 13″ N/89° 51′ 7″ W). On March 29, 2010, we collected 9 *L. sphenocephalus* clutches. Eggs were transported to the laboratory at the University of Memphis, Memphis, Tennessee. After hatching, tadpoles were maintained in 8 L aquaria in 4 L of water at a density of 2 clutches/aquaria. All tanks were the same size and dimensions and filled with the same amount of water (one tank contained a single clutch and was filled with half the amount of water for control purposes). Upon reaching the free-swimming stage (Gosner 25 [33]), tadpoles were combined from the different clutches and redistributed into tanks where density, tank size, water volume, and amount of and type of food (Tetramin® fish food) was controlled for and standardized. Such steps were used to distribute potential genetic effects of the traits measured. Test subjects were then randomly selected from this stock and placed into 1.5 L plastic containers filled with 1 L of aged tap water. Throughout the experiments, tadpoles were maintained on a 12 h light: 12 h dark photoperiod at 19°C and fed every 3 days.

### Batrachochytrium Dendrobatidis Inoculation

The *Bd* isolate used in our experiment was locally isolated from an infected adult *L. sphenocephalus* captured from the University of Memphis Biological Field Station at Meeman-Shelby State Park, Shelby County TN [35°23′22.66″N 90°02′15.75″W]) in May 2010. The isolate was grown in the laboratory in tryptone broth (1.6% tryptone, 0.2% gelatin hydrolysate, and 0.4% lactose [TGhL]) according to standard protocol [34]. Stock cultures were transferred monthly and all *Bd* inoculates were taken from these cultures. This strain has resulted in successful infections in both laboratory and field experiments.

*Bd* zoospores were harvested by adding 10.0 mL of sterile water to cultures and collecting the zoospores that emerged from the zoosporangia after 45 minutes. At Gosner 25, tadpoles were split into two groups: *Bd-*exposed and non-exposed (control) groups. The *Bd-*exposed group (N = 20) was inoculated with *Bd* through exposure to water baths containing infectious concentrations of fungal zoospores. Tadpoles were placed in individual 50 mL water baths (3 individuals per 50 mL) and an infectious concentration of zoospores (320,000 zoospores/mL) was added to each bath for 48 hours. The non-exposed group followed the same protocol but the water was added to plates with TGhL alone, thereby the additional group (N = 20) was exposed to water baths with no *Bd* zoospores. This design simulates transmission by water, a possible mode of *Bd* transmission in natural environments [35], and has resulted in successful infections in previous studies [19], [20]. After 48 hrs of exposure, *Bd*+ and *Bd*− subjects were removed from water baths and all subjects from each treatment group were placed into separate 8 L containers. After six days, 10 tadpoles for *Bd* and 10 for TM*Bd* treatments were haphazardly selected from the single *Bd*+ pool for the experiment. A similar process was carried out for subjects in control and TM treatments: were selected from the single *Bd*− pool.

### Pesticide Application

The experimental design employed 4 treatments with 10 replicates per treatment. Treatments were as follows: *Bd−* control (water), *Bd*+ control, TM+*Bd*+, and TM+*Bd*−. Individuals in TM+*Bd+* and TM+*Bd−* treatments were exposed to the same dosage of TM at a concentration of 0.6 mg/L. This concentration was chosen because it is lower than LC_50_ levels and represents a realistic estimate in situations with direct overspray [10]. Pesticide was mixed with aged tap water in bulk to achieve the respective concentration. At this point, test subjects were exposed to their respective treatment.

Water was changed every 3 days, at which time the pesticide was reapplied and individuals were fed. Pesticide concentrations were confirmed via high-pressure liquid chromatography through Pacific Agricultural Labs in Portland OR.

### Measurement of Life History Traits

We were interested in the effects of *Bd* and TM on life history traits as larvae and metamorphs. Thus, we measured life history traits of larvae prior to metamorphosis. On day 60, all larvae were anesthetized and measures of mass and snout-vent length (SVL) were recorded. Larval measurements were taken once during ontogeny to reduce the possibility of stress-induced behavioral or morphological alterations or death from repeat anesthetization and handling. When larvae began to metamorphose (day 78), containers were monitored daily for metamorphic animals. Metamorphosis was defined by the emergence of one forelimb [33]. Upon tail resorption, animals were weighed and SVL measurements were recorded.

### Batrachochytrium Dendrobatidis *qPCR Confirmation*

Infection status (*Bd*+/−) of all experimental animals was confirmed using real-time quantitative polymerase chain reaction (qPCR) following the method used by Boyle et al. [36]. DNA was extracted from cotton swabs of tadpole mouthparts taken immediately after life history measurements were taken (day 60). Swabbing tadpoles requires the removal of the tadpole from the aquatic environment by netting, holding the tadpole in hand, and twisting a swab around the tadpole’s mouthparts; thus, swabbing was conducted at a single time point to reduce handling time and stress, potentially resulting in tadpole mortality. A different pair of nitrile gloves was used between each subject to prevent contamination. Moreover, the same exposure protocols have resulted in successful infections in previous experiments (e.g. [19], [20]).

All samples were stored in 100% EtOH until qPCR analyses. Standards were obtained from CSIRO labs in Australia and were the same as those used in Boyle et al. [36]. The standards served as the positive controls and each plate contained a negative control (which tested negative on all plates). For calculations of prevalence, swabs were categorized as *Bd*-positive when zoospore equivalents were ≥1 (as used by [37], [38]).

### Statistical Analysis

Multivariate analysis of variance (MANOVA) was conducted to consider whether the *Bd* and TM had a significant effect on each dependent variable when the two treatments were considered simultaneously. We then used two-way analyses of variance (ANOVA) to test for an effect of *Bd* and TM on each response (larval mass, larval SVL, metamorphic mass, and metamorphic SVL).

## Results

No tadpoles from our control, TM+*Bd−*, or TM+*Bd+* treatments tested positive for *Bd* infection. All tadpoles from our *Bd+* treatment tested positive for infection. From qPCR in the *Bd*+ group, the mean zoospore equivalents were 168.44 (±18.44) with a range of 42.24 to 397.77.

**Figure 1:**
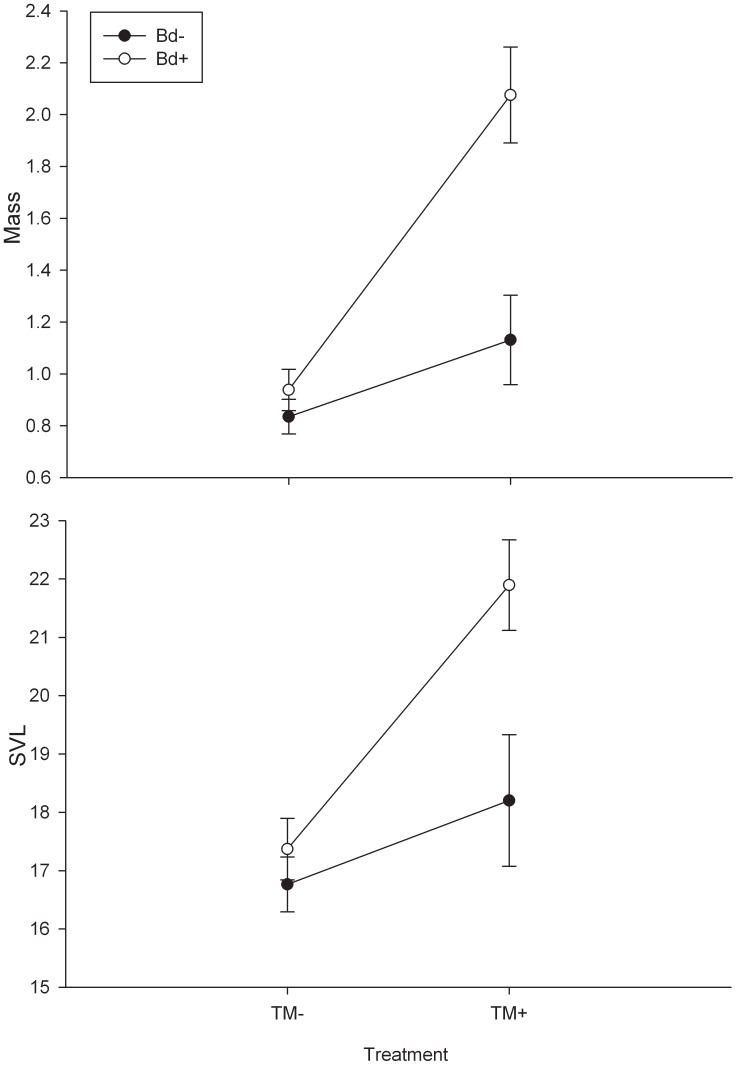
The effects of *Bd* and TM on larval mass and SVL at day 60. Asterisks (*) above plots indicate significant differences from TM− treatments (P<0.05). *indicates significant difference from TM− treatments while **indicates significant difference from TM− and *Bd−*TM+ treatments.

MANOVA indicated that there was a significant effect of *Bd* (F_2,34_ = 6.96, P = 0.003), TM (F_2,34_ = 9.53, P<0.001), and TM×*Bd* (F_2,34_ = 3.91, P = 0.030) on larval mass and SVL when considered simultaneously. There was a significant effect of TM at day 60 on larval mass (F_3,35_ = 18.63, P = <0.001) and SVL (F_3,35_ = 16.62, P = <0.001. Individuals exposed to TM alone were heavier and larger compared to *Bd* and control treatments (Fig. 1). *Bd* also had a significant effect on larval mass (F_3,35_ = 10.69, P = 0.002) and SVL (F_3,35_ = 7.71, P = 0.009) with *Bd*+ individuals being larger and longer than non-*Bd* subjects (Fig. 1). However, the presence of TM likely influenced these results. Additionally, there was a significant TM by *Bd* interaction on larval mass (F_3,35_ = 6.37, P = 0.016) and SVL (F_3,35_ = 4.45, P = 0.042. The TM×*Bd* interaction caused individuals to be heavier and larger compared to all other treatments (Fig. 1).

**Figure 2:**
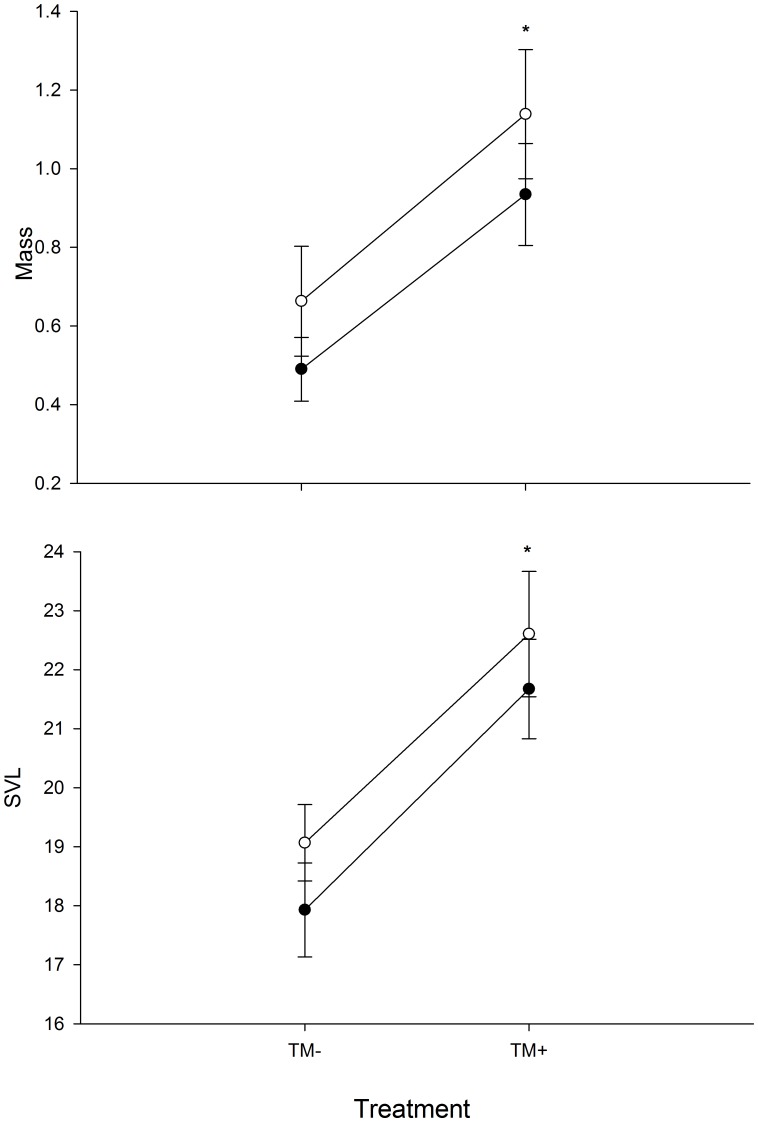
The effects of *Bd* and TM on metamorphlic mass and SVL. Asterisks (*) above plots indicate significant differences from TM− treatments (P<0.05).

MANOVA indicated that there was a significant effect of TM (F_2,25_ = 8.81, P = 0.001), but not of *Bd* (F_2,25_ = 0.88, P = 0.428) or TM×*Bd* (F_2,25_ = 0.06, P = 0.946) on metamorphic mass and SVL when considered simultaneously. TM had a significant effect on all metamorphic features as well. TM affected mass at metamorphosis (F_3, 25_ = 14.18, P = <0.009) and SVL at metamorphosis (F_3, 25_ = 10.02, P = <0.001) (Fig. 2). Independent of *Bd*, individuals subjected to TM were heavier and larger (Fig. 2) compared to all other treatments. There was not a significant effect of *Bd* on metamorphic mass (F_3, 25_ = 2.47, P = 0.128) or SVL (F_3, 25_ = 2.33, 0.1388). The TM×*Bd* interaction had similar effects as *Bd* alone. The TM×*Bd* interaction did not affect metamorphic mass (F_3, 25_ = 0.11, P = 0.7415) and metamorphic SVL (F_3, 25_ = 0.10, P = 0.759).

## Discussion

Numerous anthropogenic factors have been implicated in amphibian declines [39]–[41]. For example, amphibian trade and land use changes have facilitated the spread of *Bd* [42]. The emergence of *Bd* in areas of pesticide exposure in the forms of runoff, spraydrift, and direct overspray (personal observation) has complicated our understanding of the role of this disease in the declines. It is likely that these two factors interact to impact amphibians by altering behavior, morphology, and physiology. Although studies that have examined each perturbation have shown that the separate effects of pesticides and *Bd* are usually deleterious, the results of our study provide evidence to the contrary.

Overall, TM was advantageous to all measured traits. TM facilitated larval growth, as individuals were heavier and larger. To the best of our knowledge, ours is the first study that found a pesticide to promote such measures without obvious tradeoffs. Although other studies have found pesticides to benefit specific life history traits (e.g. growth or development), such benefits have invariably been accompanied by a tradeoff in which other trait(s) were negatively impacted. For example, Boone et al. [43] found that Woodhouse’s toads (*Bufo woodhousii*) that were exposed to carbaryl experienced increased growth at the cost of a longer developmental period. Semlitch et al. [44] found similar patterns in Gray treefrogs (*Hyla versicolor)*, in which larvae in low-density treatments exposed to the insecticide carbaryl completed metamorphosis sooner, but were smaller than those in high-density treatments. There likely are tradeoffs that exist from TM exposure, but we were not able to identify any of these in the factors typical with previous work.

It should be noted that while we controlled for developmental stage at the beginning of the experiment, starting mass and size were not measured. However, the use of development stage as a starting measure in tadpole experiments that assess growth and development through development is an experimental standard [45]–[52]. Additionally, in the presence of constant conditions, tadpole growth and development are closely correlated (for review see [53]). While studies have shown that tradeoffs occur between tadpole growth and development, individuals housed in identical conditions experience such tradeoffs together [54], [55]. Because the subjects in our study were housed in identical conditions, we are confident that initial mass and size were constant at the start by initiating the experiment with subjects at Gosner 25 [33].

Although we found no effect of *Bd* on any measured trait, qPCR revealed that exposure techniques were successful. Interestingly, larvae in *Bd* × TM treatments were heavier and larger than those in all other treatments. Additionally, larvae in this treatment that were exposed to *Bd* at the onset of the experiment tested negative for *Bd* infection via qPCR immediately prior to metamorphosis. While we assert that TM was responsible for such observations, the possibility of alterative explanations cannot be ruled out. Because we did not swab tadpoles within one week of initial exposure, it is possible that those in TM*Bd* treatments were not initially infected. However, this is extremely unlikely. Subjects in solely *Bd+* treatments all tested positive for *Bd*. It is critical to reiterate that animals in both *Bd*+ and TM*Bd*+ treatments were derived from the same infection protocols. As previously stated, tadpoles in *Bd* and TM*Bd* treatments were selected from a single pool of *Bd* exposed individuals. Because of the 100% infection success in *Bd* groups and the 0% infection in TM*Bd* groups, as well as the expose to TM after *Bd* exposure, we conclude that our infection protocols were successful in TM*Bd* subjects and TM cleared *Bd* infection in hosts.

Perhaps the most surprising finding of our study was the facilitative qualities of TM. Whereas one might propose that this fungicide could be beneficial when combined with *Bd* (i.e., by controlling the progression of the infection), it is more difficult to understand the beneficial effects of TM when administered alone. To date, no other pesticide has been found to promote life history traits such as mass and size without any obvious costs. Thus, we hypothesize multiple pathways through which such facilitations may have occurred.

Contaminant-induced alterations in amphibian growth have been well documented (for review see [56]). Specifically, aquatic contaminants have been shown to alter amphibian physiology and subsequent growth [57]–[59]. The physiology of larval growth and development, ending with metamorphosis, is regulated primarily by the hypothalamus-pituitary-thyroid (HPT) axis. Amphibian growth and development is regulated through the production of thyroid hormones (TH [60], [61]). Specifically, the thyroid hormone thyroxine (T_4_) is converted to triiodothyronine (T_3_) that acts on target tissues to promote growth and development [62]. While some argue that not all larval growth and development is controlled by TH and the HPT axis (e.g. growth hormone, prolactin [63]), other experiments manipulating TH in larvae have altered the timing of metamorphosis [58].

In our experiment, all larvae exposed to TM grew larger and weighed more than unexposed individuals. Given that the both growth in mass and size increased, we conclude that TM may have a direct effect on larval TH production, thereby increasing growth. Such effects have been observed in other environmental pollutants. The bactericide triclosan increased developmental and growth rates in larvae of bullfrogs (*Rana catesbiana* [64], [65]) and common frogs (*Rana temporaria* [66]), respectively. Studies have also shown that the pesticide acetochlor increased developmental rates in northern leopard frogs (*Rana pipiens*) and African clawed frog larvae [67], [68] by increasing T_3_ and both TH receptor levels (α, β). TM is likely acting on individuals in a similar manner to increase growth in our current study.

In addition to the possible impacts of TM on hormone concentrations, the fungicide also cleared *Bd* infection in *Bd*+ individuals. Unexpectedly, larvae exposed to *Bd* and TM were heavier and larger than those exposed to TM alone. One possible explanation for this observation could be adjusted from the “thrifty metabolism” hypothesis [69], [70]. The hypothesis holds that if an individual is malnourished early in development, their metabolism will overcompensate and, as a side effect, induce adverse health effects later in life. In such studies, the metabolism of malnourished young overcompensated upon release from stressful circumstances, and this resulted in ‘over-nutrition’ (or catch-up weigh gain) with subsequent health issues such as diabetes and obesity. While we did not test the effects of *Bd* or TM on foraging abilities, is it possible that this hypothesis could apply to our data. We hypothesize that individuals exposed to both TM and *Bd* were forced to initially cope with the deleterious effects of *Bd*. However, upon the clearing of *Bd* by TM, these newly uninfected individuals may have overcompensated by increasing feeding rates. This hypothesis parallels the “enemy release” hypothesis [28], [71]. Usually pertaining to invasive species, the invasive organism is placed into a naïve habitat without any predators to control population sizes. Because of this ‘release,’ the introduced organism can thrive and usually become a pest in the absence of any predators. In our case, TM is “releasing” infected tadpoles from *Bd*; thus, allowing newly uninfected individuals to overcompensate in the absence of a previous health threat. This, combined with the beneficial effects of TM, might have resulted in growth that surpassed all other treatments.

While our study is the first to show significant promotions in life history traits of *Bd−* and pesticide-exposed amphibians, Gahl et al. [72] recently showed similar trends in *Bd*− and glyphosate-exposed frogs. In the study, exposure to *Bd* or glyphosate alone did not significantly alter growth. However, in both *Bd-*exposed and unexposed, a trend was observed with individuals exposed to glyphosate being larger and heavier than those not exposed to the pesticide. They cited three possibilities for their observations: possible direct inhibition of *Bd* in water, the addition of nutrients to the system from glyphosate, and glyphosate-induced immune responses that would operate to fight *Bd* infection. These results corroborate our current findings where in both *Bd-*exposed and unexposed anurans, the addition of TM into the system facilitated growth compared to subjects in treatments without pesticides.

The results of our current study were unexpected. While we predicted that TM would clear infection in *Bd* exposed individuals, we did not predict the overall morphologically beneficial properties of TM to both *Bd+* and *Bd−* individuals. We have offered reasonable speculations as to the mechanism whereby such benefits might occur, though we reiterate that they are just that – speculations. It is clear that further research must be conducted to elucidate the pathways through which TM is acting upon larvae to induce growth in both larvae and adults. Additionally, researchers must work to make clear possible effects of such substances (i.e., effects on reproduction and fitness). Only through such research will we truly be able to assess the effects of such contaminants and disease on amphibian health.
